# Natural Scaffolds with Multi-Target Activity for the Potential Treatment of Alzheimer’s Disease

**DOI:** 10.3390/molecules23092182

**Published:** 2018-08-29

**Authors:** Luca Piemontese, Gabriele Vitucci, Marco Catto, Antonio Laghezza, Filippo Maria Perna, Mariagrazia Rullo, Fulvio Loiodice, Vito Capriati, Michele Solfrizzo

**Affiliations:** 1 Dipartimento di Farmacia-Scienze del Farmaco, Università degli Studi di Bari “Aldo Moro”, Via E. Orabona 4, 70125 Bari, Italy; marco.catto@uniba.it (M.C.); antonio.laghezza@uniba.it (A.L.); filippo.perna@uniba.it (F.M.P.); mariagrazia.rullo@uniba.it (M.R.); fulvio.loiodice@uniba.it (F.L.); vito.capriati@uniba.it (V.C.); 2 Consiglio Nazionale delle Ricerche-Istituto di Scienze delle Produzioni Alimentari (CNR-ISPA), via Amendola, 122/O, 70125 Bari, Italy; gabrielevitucci@gmail.com (G.V.); michele.solfrizzo@ispa.cnr.it (M.S.); 3 Consortium C.I.N.M.P.I.S., Via E. Orabona 4, 70125 Bari, Italy

**Keywords:** bioactive natural compounds, secondary metabolites, Alzheimer’s disease

## Abstract

A few symptomatic drugs are currently available for Alzheimer’s Disease (AD) therapy, but these molecules are only able to temporary improve the cognitive capacity of the patients if administered in the first stages of the pathology. Recently, important advances have been achieved about the knowledge of this complex condition, which is now considered a multi-factorial disease. Researchers are, thus, more oriented toward the preparation of molecules being able to contemporaneously act on different pathological features. To date, the inhibition of acetylcholinesterase (AChE) and of β-amyloid (Aβ) aggregation as well as the antioxidant activity and the removal and/or redistribution of metal ions at the level of the nervous system are the most common investigated targets for the treatment of AD. Since many natural compounds show multiple biological properties, a series of secondary metabolites of plants or fungi with suitable structural characteristics have been selected and assayed in order to evaluate their potential role in the preparation of multi-target agents. Out of six compounds evaluated, **1** showed the best activity as an antioxidant (EC_50_ = 2.6 ± 0.2 μmol/µmol of DPPH) while compound **2** proved to be effective in the inhibition of AChE (IC_50_ = 6.86 ± 0.67 μM) and Aβ_1–40_ aggregation (IC_50_ = 74 ± 1 μM). Furthermore, compound **6** inhibited BChE (IC_50_ = 1.75 ± 0.59 μM) with a good selectivity toward AChE (IC_50_ = 86.0 ± 15.0 μM). Moreover, preliminary tests on metal chelation suggested a possible interaction between compounds **1, 3** and **4** and copper (II). Molecules with the best multi-target profiles will be used as starting hit compounds to appropriately address future studies of Structure-Activity Relationships (SARs).

## 1. Introduction

Alzheimer’s Disease (AD) is a neurodegenerative pathology first described by Aloïs Alzheimer in 1907 as an “unusual illness of the cerebral cortex” [1]. Currently, it is recognized as a real social and economic issue. The average annual cost is estimated as $15,000–20,000 for each patient [2] and the incidence is currently 34/1000 persons >60 years old with 42.1% of prevalence at >95 years of age [2,3,4,5]. Based on these data, the impact of the pathology is expected to be devastating in the near future, assuming an increase of life expectancy even in Third World countries. In the absence of new therapies able to prevent or treat such a pathology, it is estimated that the number of people with dementia will reach more than 130 million by 2050 [6]. 

The main problem connected with AD is the absolute lack of effective treatments. In the last several years, many routes have been suggested for understanding the pathogenesis and addressing the relevant drug strategies to fight this neurodegenerative disease. The most common pursued hypotheses are the cholinergic and the amyloid ones [7]. 

Numerous research studies link the damage of cholinergic neurons with the onset of the pathology [8]. According to these considerations, four of the five symptomatic drugs that have been used for AD therapies, are AChE inhibitors (AChEIs). These molecules known as Donepezil, Rivastigmine, and Galantamine, and Tacrine (the first one approved in 1993 but now withdrawn from the market due to its toxic effects) [9] are only able to temporarily improve the cognitive skills of the patients. 

In addition, the hydrolytic enzyme acetylcholinesterase (AChE) was proven recently to play a certain role in several secondary non-cholinergic functions and in the deposition of amyloid peptides (Aβ) in the extracellular environment of the brain, which was reported in several AD diagnosed patients [8]. The Aβ peptides are produced by the cleavage of the membrane-anchored APP (β-Amyloid Precursor Protein) in the inter-synaptic environment operated by secretases and are involved in the formation of the so-called amyloid plaques [10]. These complexes include in their structures heavy metals such as copper (II) and zinc (II) [11,12,13,14]. Their cytotoxicity has been associated by several authors with the production of oxygen radicals (ROS) and consequent neuronal inflammation and degeneration [10,15]. 

In the last decade, most research groups focused their activities on the synthesis of multi-target agents with multiple actions to face the classical features recognized as important at the onset of AD. They aimed to improve the therapeutic efficacy by using synergistic actions. To date, NMDA receptor antagonism as well as the inhibition of cholinesterases (ChEs) and beta-Secretase (BACE), inhibition of Abeta amyloid plaques (Aβ) aggregation, and antioxidant activity are the most common investigated targets. The chelation of heavy metal cations has also been the subject of several research studies [11,12,13,14]. Moreover, numerous clinical studies have been recently focused on the repositioning of old drugs such as PPAR agonists [16], which are already used in the therapy of atherosclerosis and diabetes [17,18]. Particularly appealing is the use of natural compounds [19] in food supplements especially at the industrial level [20], which are also a source of inspiration for the synthesis of molecules with multi-target activity [19,20]. 

With the aim to discover new biological activities associated with natural compounds, five secondary fungal metabolites (**1**–**6**) and one plant metabolite (**4**) with suitable structural characteristics (i.e., low molecular weight, heterocyclic moieties, and particular substituents such as hydroxyl groups) have been selected in this work and assayed for a preliminary evaluation of their potential as new scaffolds for the design and synthesis of new multi-target ligands useful for the treatment of AD. We choose these molecules on the basis of the consideration that coumarin-like nuclei are frequently used in the synthesis of AChE inhibitors [21]. In addition, heterocyclic scaffolds with appropriate substituents have been reported as antioxidants or copper/zinc/iron chelators and planar structures are able to, in general, block the Aβ aggregation [9,20]. 

Consequently, we executed assays on the ChEs activities (AChE and BChE inhibition using a modified protocol of Ellman’s spectrophotometric assay adapted to a 96-well plate system), on the antioxidant effect by the DPPH (2,2-diphenyl-1-picrylhydrazyl) method and on Aβ aggregation inhibition using a spectrofluorimetric assay (measuring ThT fluorescence in the presence of the peptide). A preliminary evaluation of the interaction of these compounds with copper (II) and zinc (II) ions through spectrophotometric measures was performed as well. The technical approaches are described below (see Section 3, Materials and Methods). Our aim is to search for an innovative therapeutic intervention that should address both the limitation ofAChE enzyme activity and the inhibition of the aggregation of Aβ peptides. Moreover, sequestering heavy metals such as copper (II) can be useful in order to prevent the production of ROS and inhibit the formation of amyloid plaques as well.

**Figure 1:**
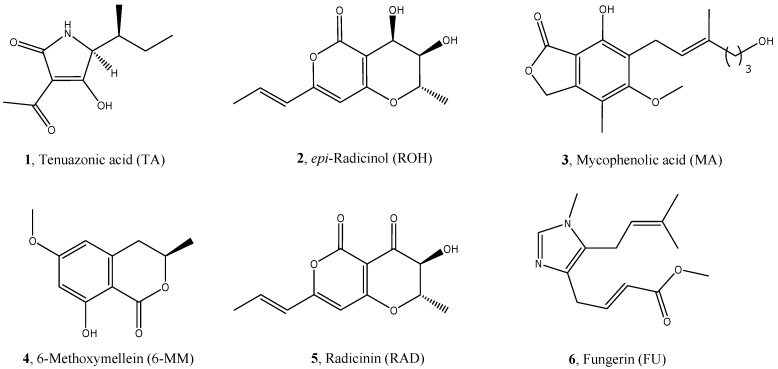
Chemical structures of the selected natural compounds.

The structures of the selected compounds are depicted in Figure 1. All these molecules have been isolated and characterized over the last three decades, but studies about their physiological role and biological activity are still lacking with the exception of compound **6** for which anti-AChE activity was reported [22].

In detail, Tenuazonic acid (**1**, TA) is a secondary metabolite produced mainly by fungi belonging to the *Alternaria* genera. It can be found in soil, decaying organic vegetable matter, and in both cultivated and non-cultivated plants. It has been isolated from fruits, vegetables, cereals, oilseeds, edible nuts, and beans. It is a colorless oil, soluble in chloroform and methanol, and usually stored as copper salt. Tenuazonic acid is toxic to a wide range of plants, fungi, bacteria, and viruses and it is known to be a phytotoxin [23]. 2-*epi*-Radicinol (**2**, ROH) is a secondary metabolite produced by *Alternaria radicina* grown on carrots and is reported as a phytotoxic compound because it reduces root elongation of germinating carrot seeds when tested on a laboratory scale [23]. Like other fungal metabolites with similar chemical structures and produced by several fungi of the *Alternaria* genera, it is not hazardous for consumers [24]. Mycophenolic acid (**3**, MA) is a fungal metabolite that was discovered by Bartolomeo Gosio in 1893 as an antibiotic against *Bacillus anthracis*. It is active as an immunosuppressant drug and is a potent anti-proliferative usually used as part of triple therapy after renal transplantation including a calcineurin inhibitor (ciclosporin or tacrolimus) and prednisolone [25]. It also possesses antiviral, antifungal, and anti-psoriasis activities [26]. 6-Methoxymellein (**4**, 6-MM) is a phytoalexin with a dihydro-isocoumarin skeleton, which accumulates in carrots and is associated with the bitterness in strained carrots and is, in part, responsible for the sensory quality of these vegetables. The production of 6-MM in carrots and carrot cell suspensions has also been reported in response to either infection by fungi or treatment with abiotic elicitors [27,28]. Radicinin (**5**, RAD) is produced by *Alternaria radicina*, which is a seed borne fungal pathogen responsible for the black rot disease of carrots. This molecule is classified as a phytotoxin with antifungal, antibiotic, insecticidal, and plant growth regulatory activities [27,28]. Recently, it has been reported that RAD inhibits *Xylella fastidiosa*, which is the causal agent of Pierce’s Disease of grapevine and other plants [29]. Visoltricin is an imidazolic biologically active metabolite produced by *Fusarium tricinctum* that was discovered in 1989 and reported to have anticholinesterase activity, toxicity in the *Artemia salina* test, cytotoxicity against human tumor cell lines, and a miotic effect on rabbit eyes [18,30,31]. Its structure was successively and slightly revised because it is identical to Fungerin (**6**, FU), which is an antifungal metabolite independently isolated from a culture of a strain of *Fusarium* sp. [32]. Recently, Fungerin has been reported to inhibit the polymerization of microtubules interrupting the cell cycle in the M-phase [33]. 

## 2. Results and Discussion

Compound **5**, which is produced and isolated in this study from rice cultures of *A. radicina* [23,34], has been identified as Radicinin by using LC-Q-TOF mass spectrometry and by comparing the ^1^H and ^13^C-NMR results with those reported in the literature [35]. Compounds **1** to **6** were evaluated for the AChE and BChE inhibition activity using an enzymatic assay. The antioxidant capacity was assessed using the DPPH radical scavenging activity assay while the anti-amyloidogenic activity was determined by in vitro assays in order to quantify the inhibition of the aggregation of the Aβ_1–40_. Moreover, considering that many natural compounds are able to chelate metals, a fast preliminary test using UV spectrophotometry was arranged in order to evaluate the interaction of some compounds, which are selected on the basis of their chemical structures, with Copper (II) and Zinc (II) at the physiological pH. The experimental conditions are reported in Section 3. Clioquinol was tested as a reference compound on the basis of its structural characteristics (molecular weight, heterocyclic structure) and its biological activity. This molecule was recently used in clinical trials for the treatment of AD on the basis of its marked ability in chelating heavy metals [36,37]. Moreover, we found a multi-target activity in our experimental conditions, which was already reported in past papers [38]. Galantamine, Gallic acid, and Quercetin were used as golden standards for the ChEs inhibition activity [39], antioxidant activity, and inhibition of the aggregation of the Aβ_1–40_, respectively [39]. The results are reported in Table 1.

As mentioned above, except for compound **6** whose activity as an AChE inhibitor was already reported in the literature in medium-high micromolar range [22], no data are available for the other compounds even if natural and synthetic coumarin-like compounds have been widely reported as potential nuclei involved in the inhibition of AChE [21]. In fact, it is demonstrated that their ability to interact with the Peripheral Active Site (PAS) of the enzyme is crucial in the mechanism of the action of cholinesterase inhibitors [21]. Therefore, it is not surprising the good activity of compounds **2**–**5**, which show an IC_50_ in the low micromolar range (6.86 to 11.4 µM) without a significant difference between each other. These data are comparable with those recently reported by Ali et al. about the activities of umbelliferone (AChEi as means ± SEMs of triplicate experiments =105.48 ± 0.57 µM), 6-formyl-umbelliferone (16.70 ± 1.62 µM), and 8-formyl-umbelliferon (19.13 ± 0.57 µM) isolated from *Artemisia decursiva* [40]. 

Unlike other secondary metabolites produced by *Alternaria* species and in particular Altenuene [41], Tenuazonic Acid (compound **1**) showed a marked inhibition of AChE (8.13 ± 0.08 μM) with a weak activity on the other tested cholinesterase (7% of inhibition at 10μM). Altenuene was purified by Bhagat et al. from a culture of endophytic fungi isolated from *V. rosea* (*Catharanthus roseus*). The authors did not report the inhibitory effect of the single molecule. However, they attributed to Altenuene the anticholinesterase effect of the extract in the isolate VS-10 (78% for AChE and 73% for BChE) in the condition described for the screening assay [41]. In our study, the only molecule with a significant effect also on BChE was Fungerin (compound 6, IC_50_ = 1.75 ± 0.59 μM) with a potency as high as about 50 times compared to that on AChE, which is five times better than the reference compound Galantamine. This result is really interesting considering that, in addition to AChE, BChE as well plays an important role in the cholinergic neurotransmission [8,42] in the central nervous system (CNS). In addition, recent studies suggest that an unselective ChE inhibitor should lead to better clinical results [8,43]. Our data can be compared with those reported for pteryxin, which is a dihydropyranocoumarin derivative found in the *Apiaceae* family [44]. This natural molecule was tested in vitro on cholinesterases using an ELISA microplate reader at 100 μg/mL. No data about IC_50_ were reported. However, on the basis of the percentage of inhibition (9.30 ± 1.86% and 91.62 ± 1.53% against AChE and BChE, respectively), the authors concluded that pterixyn is a strong BChE inhibitor and one better than Galantamine (81.93 ± 2.52% of inhibition at 100 μg/mL) [44]. Therefore, like compound **1**, it can be considered as a lead compound to develop novel BChE inhibitors for AD treatment [44]. 

All the tested compounds (**1**–**6**) revealed a certain activity as inhibitors of Aβ_1–40_ aggregation at 100 μM. These results confirm that heterocyclic condensed rings can exert a disturbing action in forming these protein aggregates and may be associated with the disruption of the conformation in β-sheets, which was previously reported [45]. The best activity was registered for *epi*-radicinol (compound **2**), but compounds **1** (tenuazonic acid) and **4** (6-methoxymellein) showed an IC_50_ close to 100 μM. This is far from the clioquinol (7.6 ± 0.8 μM) and quercetin (0.82 ± 0.07 μM) but is really promising considering the possibility of chemical functionalization of the structures in future structure-activity relationship (SAR) studies. 

Other studies about natural compounds with this kind of biological activity have been reported in literature in the recent past. In particular, some derivatives of resveratrol (scirpusin A and ε-viniferin glucoside) have been described as potential therapeutic agents in treating AD due to their strong inhibitory activity of Aβ aggregation (IC_50_ were 0.7 ± 0.3 μM for scirpusin A and 0.2 ± 0.3 μM for ε-viniferin glucoside) [46]. However, the authors concluded that the efficacy and utility of these molecules will depend on their bioavailability in vivo [46]. This is a big issue for this type of structure. This is really different from those selected in our study and less suitable for derivatization due to their higher polarity and molecular weight [9]. 

Anthoxanthin polyphenols have been studied as well for their ability to reduce a Aβ oligomer-induced neuronal response [47]. These molecules and in particular Kaempferol (KAE) have been demonstrated to act with a dual synergic mechanism through modulation of oligomerization and antioxidant activity [47], which is itself an important factor in the Aβ neurotoxicity [48,49]. 

The ability of inhibiting ROS accumulation of KAE is not surprising. In fact, it is widely recognized as typical of numerous natural metabolites including a polyphenolic skeleton in their chemical structures [47,48,50]. 

**Figure 2:**
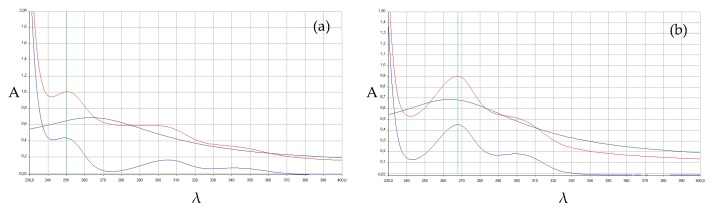
UV spectra of copper (II) solution (green track), ligand solution (blue track), and copper (II)/ligand 4:1 solution (red track). The experimental conditions are reported in Section 4.5. (**a**) compound **3** and (**b**) compound **4**. A = absorbance, λ = wavelength (nm).

As predicted on the basis of their structural features, a couple of our selected compounds and in particular Tenuazonic Acid (**1**) and Mycophenolic Acid (**3**) were demonstrated to have a significant antioxidant effect with 2.6 and 14.7 μmol/µmol of DPPH, respectively. This biological effect and the particular structure prompted us to investigate the ability of these two compounds to chelate heavy metals. The results of preliminary tests on metal interaction proved to be particularly interesting. Both molecules, in fact, showed a characteristic UV spectrum in solution in the presence of a copper (II) salt. In particular, the absorbance in each point of the curve was slightly different from the sum of the absorbance displayed by the spectra of the ligand and copper salt alone (Figure 2a and Figure S1). This behavior is similar to that of the well-known chelating compound clioquinol under the same experimental conditions (Figure S1) and, therefore, it might reasonably confirm a possible interaction between the ligand and the metal. 6-Methoxymellein (**4**) showed a similar effect (Figure 2b) while, apart from clioquinol, noone of these compounds was able to interact with zinc cations (Figure S2).

Considering that, when exploring multi-target ligands, the activities are not expected to be very high on each target, at least in the preliminary stage of research, the potency of our selected and tested compounds (in particular **1** and **3**) in the low-medium micromolar range can be considered as a good result. Among the already mentioned works, Ali et al. tested their compounds on BChE and BACE1 with a good inhibitory effect in particular for 6-formyl-umbelliferone [40]. For both research groups, these results will be the starting point for exploring the possibility to increase the activities and obtain new and more efficient chemical entities, through studies of Structure-Activity Relationships (SARs). 

Moreover, several recent studies reported the multi-target activity of molecules that include natural-inspired scaffolds in their complex chemical structures (reviewed by Hiremathad [20] and Jalili-Baleh et al. [51]). The results obtained in our preliminary screening encourage us to use compounds **1** to **6** to design new potential drugs with better pharmacological profiles. In fact, the low molecular weight and the presence of reactive residues in their chemical structures give us the possibility to combine the most interesting scaffolds with other nuclei (e.g., with NMDA antagonistic action), according to the classical strategies of multi-target drugs synthesis [20,38,39,52]. 

One important issue in the research of new drugs active on CNS is the ability of the molecules to cross the blood-brain-barrier (BBB) [9,45]. This preliminary study was focused on the selection of scaffolds to be used as a hit compound in the research of new more complex chemical entities. Therefore, at this step of our work, any speculation about the use in therapy is not useful. However, all the selected compounds have low molecular weight and very low or absolutely no water solubility. For these reasons, they have a good chance to pass BBB [9,53]. 

## 3. Materials and Methods 

Compounds **1**, **2**, **4**, and **6** were previously produced, isolated, and characterized in the CNR-ISPA laboratories, according to the literature [23,27,30,31], and made available by the M.S. Compound **5**, which was produced, isolated, and identified in this study using a previously reported method [21,34] with some modifications (Section 3.1). Compound **3** is commercially available, was purchased from Sigma-Aldrich, (Milan, Italy), and used for the tests without any further purification. 

### 3.1. Production, Isolation, and Identification of Radicinin

*Alternaria radicina* (isolate ITEM 4218, from CNR-ISPA fungal culture collection) was grown on 20 g aliquots of rice kernels. In particular, 20 g of rice kernels were moistened with 10 mL of distilled water in 250 mL Erlenmeyer flasks and autoclaved at 121 °C for 20 min. The fungal cultures were incubated at 28 °C for 21 days in the darkness. Then, the rice fungal cultures were combined, dried at 40 °C, finely grounded with a blender, and aliquots of 20 g were extracted with 80 mL of a mixture of acetonitrile:methanol:water (45:10:45, *v*/*v*/*v*) at a pH of 3 (HCl) for 30 min by shaking. After filtration, the solution was liquid-liquid extracted with 3 × 50 mL of CH_2_Cl_2_ in a separatory funnel. The procedure was performed on 11 aliquots of 20 g. The combined organic portions were dried over anhydrous sodium sulfate and evaporated to dryness under a vacuum at 40 °C [23]. 

The final residue (1.7 g) was reconstituted with 10 mL of chloroform and chromatographed twice on a 22 × 2.2 cm i.d. preparative silica gel column packed with silica gel (0.063 to 0.200 mm particle size, 70 to 230 mesh) (Merck, Darmstadt, Germany) in CHCl_3_. Solvents were purchased from Carlo Erba Reagents S.r.l. (Cornaredo, Milano, Italy) and used without any further purification. The first column was eluted sequentially with 150 mL of CHCl_3_:0.1% glacial acetic acid (99:1, *v*/*v*), 200 mL of CHCl_3_:MeOH:0.1% glacial acid acetic (94:6:1, *v*/*v*/*v*), and 300 mL of CHCl_3_:MeOH:0.1% glacial acid acetic (88:12:1, *v*/*v*/*v*). Thirty fractions of 20 mL ca. each were separately collected and analyzed by glass TLC and HPLC-UV/DAD to check the presence and purity of radicinin. Glass TLC plates were silica gel coated with a fluorescent indicator F254 10 × 10 cm, 0.1 mm thickness and were purchased from Merck (Darmstadt, Germany). The compound resulted in fractions F20–23. Radicinin was tentatively identified in the HPLC chromatograms by its characteristic UV spectrum having a maximum at 345 nm. The four fractions were combined, concentrated to about 10 mL under vacuum at 40 °C, further chromatographed on a second silica gel preparative column, packed as reported above, and eluted sequentially with 100 mL of CHCl_3_:0.1% glacial acid acetic (95.5:0.5, *v*/*v*/*v*) and 500 mL of CHCl_3_:MeOH:0.1% glacial acid acetic (97.5:2:0.5, *v*/*v*/*v*). Thirty fractions of 20 mL ca. each were collected and analyzed for radicinin as reported above. Radicinin was tentatively identified in the fractions F17–F23 at a different degree of purity. 

The fractions were singularly concentrated under vacuum at 40 °C, redissolved in 0.3 mL of CHCl_3_, and finally purified by several semi-preparative glass TLC plates, silica gel coated with flourescent indicator F254 20 × 20 cm, and 0.5 mm thickness purchased from Merck (Darmstadt, Germany). TLC plates were eluted with a mobile phase solution of CHCl_3_:*n*-hexane:2-propanol:0.1% glacial acetic acid (50:30:20:1, *v*/*v*/*v*/*v*). The stripe of radicinin (Rf = 0.85) was visualized under UV light at 254 nm and the silica gel of the stripe was scraped from the plate and put in an empty mini column containing a frit. A total of 7 plates were used to purify the fractions F17–F23. Radicinin was recovered from each scraped silica gel stripe by eluting 10 mL of a mixture of chloroform: methanol (8:2 *v*/*v*). After filtration through Whatman^®^ n.4, the resulting solutions were evaporated to dryness under nitrogen stream at 40 °C, reconstituted with 1 mL of CH_3_CN, and aliquots were diluted with HPLC mobile phase and analyzed by HPLC-UV/DAD to check the purity of radicinin. The HPLC column was a Symmetry Shield C18 reversed-phase 150 × 4.6 mm i.d. 5 μm (Waters, Milford, MA, USA) and was preceded by a Rheodyne guard filter (3 mm, 0.5 μm). The mobile phase was a linear gradient of acetonitrile in water from 10% to 30% in 22 min. The flow rate was 1 mL/min. The peak of radicinin was identified in the chromatogram by its characteristic UV spectrum having the maximum of absorbance at 345 nm. A total of 8 mg of a pale yellow solid (m.p. 235 to 237 °C) of radicinin was obtained with a purity >97% as determined by ^1^H and ^13^C NMR spectra and HPLC-UV/DAD (Figures S1–S3, supporting the information file). 

The high resolution mass spectrometry (HRMS-ESI)) experiments were carried out with a hybrid Q-TOF mass spectrometer AGILENT 1100 LC/MSD equipped with an ion-spray ionization source. 

HRMS (ESI, pos) of radicinin: M=C_12_H_12_O_5_, for [M + Na]^+^ calculated: 259.0577, found: 259.0578 (Δ = −0.27), for [M + H]^+^ calculated: 237.0757, found: 237.0755 (Δ = 1.13), HRMS (ESI, neg): for [M − H]^−^ calculated: 235.0612, found: 235.0606 (Δ = 2.40).

The ^1^H and ^13^C NMR spectra were recorded on a 600 Bruker spectrometer at room temperature using CDCl_3_ as the solvent (δ = 7.26 ppm for ^1^H spectra, δ = 77.0 ppm for ^13^C spectra).

^1^H NMR (600 MHz, δ, ppm): 1.67 (d, *J* = 6.2 Hz, 3H, H_a_), 1.98 (d, *J* = 6.9 Hz, 3H, H_b_), 3.86 (s, 1H, H_c_), 4.01 (d, *J* = 12.3 Hz, 1H, H_e_), 4.35–4.40 (m, 1H, H_d_), 5.86 (s, 1H, H_f_), 6.05 (d, *J* = 15.4 Hz, 1H, H_g_), 6.96–7.02 (m, 1H, H_h_).

^13^C NMR (125 MHz, δ, ppm): 188.6 (C-1), 176.4 (C-4), 164.4, (C-7), 156.7 (C-6), 141.1 (C-10), 122.6 (C-9), 97.9 (C-8), 97.8 (C-5), 80.1 (C-3), 72.0 (C-2), 18.8 (C-11), 18.1 (C-12).

The ^1^H and ^13^C NMR spectra of purified radicinin (Figures S3 and S4, respectively) have been reported in the supporting information file, as well as chromatogram and UV spectrum (Figure S5). 

### 3.2. Inhibition of Aβ_1–40_ Aggregation

The spectrofluorimetric assays measured ThT fluorescence in the presence of Aβ and were done as previously described [39]. Co-incubation samples were prepared in 96-well black, non-binding microplates (Greiner Bio-One GmbH, Frickenhausen, Germany) by diluting Aβ_1–40_ (EZBiolab, Carmel, IN, USA) alone or in the presence of the inhibitor to a final concentration of 30 μM and 100 μM, respectively, in PBS (pH 7.4) containing 10% DMSO and 2% 1,1,1,3,3,3-hexafluoro-2-propanol (HFIP). After 2 h of incubation at 25 °C, 25 μM ThT solution in phosphate buffer (pH 6.0) was added and fluorescence was read in a multi-plate reader Infinite M1000 Pro (Tecan, Cernusco S.N., Italy). For most active compounds (inhibition > 80%), IC_50_ was determined from seven concentrations (ranging from 1 μM to 1000 μM) of the inhibitor, prepared by diluting a stock DMSO solution 10 mM with PBS. Assays were run in triplicate. Values are expressed as mean ± SEM.

### 3.3. AChE and BChE Inhibition

A modified protocol of Ellman’s spectrophotometric assay [54] adapted to a 96-well plate procedure was followed as previously described [39]. Incubation samples of AChE from electric eel or BChE from equine serum (eeAChE, 463 U/mg, and esBChE, 13 U/mg, Sigma-Aldrich, Milan, Italy) were set in phosphate buffer (pH 8.0) containing 0.5 mM 5,5′-dithiobis(2-nitrobenzoic acid) (DTNB; Sigma-Aldrich, Milan, Italy) as the chromophoric reagent alone or in the presence of the inhibitor (10 μM). Incubations were carried out in clear flat-bottomed, 96-well plates (Greiner Bio-One GmbH, Frickenhausen, Germany) in duplicate. For most active compounds (inhibition > 60%), IC_50_ was determined from seven solutions (ranging from 30 μM to 0.03 μM as the final concentrations) of inhibitor and prepared by diluting a stock DMSO solution 1000 μM with a work buffer. After incubation for 20 min at 25 °C, 0.5 mM acetyl- or butyrylthiocholine iodide (Sigma-Aldrich, Milan, Italy) were added as the substrates and AChE-catalyzed hydrolysis was followed by measuring the increase of absorbance at 412 nm for 5 min at 25 °C in a Tecan Infinite M1000 Pro multiplate reader (Tecan, Cernusco S.N., Italy). Inhibition values and IC_50_s were calculated with a GraphPad Prism as the mean of three independent experiments and are expressed as mean ± SEM. 

### 3.4. Antioxidant Activity (DPPH Method)

The DPPH assay is routinely practiced for the assessment of the free radical scavenging potential of an antioxidant molecule. EC_50_ value is defined as the amount of antioxidant necessary to decrease the absorbance of 1 µmol of DPPH by 50% of the initial absorbance. 

The DPPH radical scavenging assay was performed in 96-well microplates according to the method reported by Blois [55] with some modifications [56,57,58].

A freshly prepared solution of DPPH in methanol (100 µM final concentration) was added to test compounds methanolic solution. The mixtures were shaken vigorously and left to stand in the dark for 30 min at room temperature. Then absorbance was read at 520 nm using a spectrophotometric plate reader (Victor 3 Perkin-Elmer).

The antioxidant activity was determined as the RSA% (radical scavenging activity) and calculated using the following equation: RSA% = 100 × [(Ao − Ai)/Ao] where Ao and Ai are the DPPH absorbance in the absence or in the presence of antioxidants, respectively. Different sample concentrations were used in order to obtain anti-radical curves for calculating the EC_50_ values. Anti-radical curves were plotted referring to log concentration on the x-axis and their RSA% on the y-axis. The EC_50_ values and statistical analyses were processed using the GraphPad Prism 5 software (San Diego, CA, USA).

Values of all parameters are expressed as mean ± SEM of at least three independent measurements in triplicate.

### 3.5. Metal-Ligands Interactions 

A preliminary determination of the qualitative interactions between Copper (II) or Zinc (II) and the ligands **1**–**6** was performed following a modified protocol using a spectrophotometric assay [52]. DMSO stock solutions have been prepared for molecules at 10 μM. Subsequently, the individual stock solutions were diluted in phosphate buffer at a pH of 7.4 at a concentration of 100 nM and mixed with an equal amount of buffer solution at a pH of 7.4 of a 400 nM solution of CuSO_4_·5H_2_O or ZnCl_2_. The UV spectra of the three solutions obtained were recorded as well as those of the solutions of the two salts that were mixed with an equal amount of buffer solution at a pH of 7.4.

The UV spectra of the salt solution (200 nM), the solution of the single molecule (50 nM), and the solution of the molecule + salt (in concentration ratio 1:4) were then superimposed. If the sum of the absorbances of the first two spectra does not correspond in each point to the absorbance recorded in the third one, we supposed a probable interaction.

## 4. Conclusions

Five natural fungal secondary metabolites and one plant metabolite have been identified as possible scaffolds for the development of new potential drugs for treating AD. These molecules were tested for their biological activities on several targets such as AChE, BChE and Aβ_1–40_ aggregation inhibition, antioxidant activity, and copper (II) and zinc (II) interaction. Compound **2** resulted the best AChE and Aβ_1–40_ aggregation inhibitor with an IC_50_ in the low micromolar range while compound **6** was the only one able to inhibit both AChE and BChE. Compounds **1** and **3** showed an interesting multi-target profile that considers the antioxidant activities and the capability of interaction with copper (II). These promising results will address our future studies of Structure-Activity Relationships. 

## Figures and Tables

**Table 1:** Biological assays on compounds **1** to **6**.

	eeAChEi IC_50_ (μM ± SEM)	esBChEi IC_50_ (μM ± SEM)	Antioxidant Activity EC_50_ (μmol/µmol of DPPH ± SEM)	iAβ IC_50_ (μM ± SEM)
Galantamine	0.51 ± 0.10	8.70 ± 1.02	n.d.	n.d.
Gallic acid	n.d.	n.d.	0.054 ± 0.004	n.d.
Quercetin	n.d.	n.d.	n.d.	0.82 ± 0.07
Clioquinol	8.12 ± 1.00	%I (10μM): 10 ± 1%	0.74 ± 0.04	7.6 ± 0.8
**1**	8.13 ± 0.08	%I (10μM): 7 ± 1%	2.6 ± 0.2	%I (100μM): 50 ± 8
**2**	6.86 ± 0.67	i.a.	> 100	74 ± 1
**3**	7.84 ± 0.72	i.a.	14.7 ± 3.4	%I (100μM): 38 ± 3
**4**	11.4 ± 0.8	%I (10μM): 10 ± 3%	> 100	98 ± 3
**5**	8.96 ± 0.97	%I (10μM): 6 ± 1%	>100	%I (100μM): 44 ± 3
**6**	86.0 ± 15.0	1.75 ± 0.59	>100	%I (100μM): 33 ± 9

eeAChEi = inhibition of acetylcholinesterase from electric eel. esBChEi = inhibition of butyrylcholinesterase from equine serum. iAβ = inhibition of Aβ_1–40_ aggregation. % I = percentage of inhibition at 100 μM. i.a. = not active. n.d. = not determined.

## Supplementary Materials

The following are available online at http://www.mdpi.com/1420-3049/23/9/2182/s1, Figure S1: UV spectra of copper (II) solution, ligand solution and copper (II)/ligand 4:1 solution of clioquinol (a) and compound 1 (b), Figure S2: UV spectra of zinc (II) solution, ligand solution, and copper (II)/ligand 4:1 solution of clioquinol (a) and compound 2 (b), Figure S3: ^1^H NMR spectrum (CDCl_3_; 600 MHz) of Radicinin (purity > 97%), Figure S4: ^13^C NMR spectrum (CDCl_3_, 125 MHz) of Radicinin, Figure S5: Chromatogram and UV spectrum of Radicinin.
