# Supplementary material for: Natural Scaffolds with Multi-Target Activity for the Potential Treatment of Alzheimer’s Disease
Source: Molecules. 2018 Aug 29;23(9):2182. doi: 10.3390/molecules23092182 (PMC6225478; doi:10.3390/molecules23092182)
Supplement: Supplementary file 1 [file molecules-23-02182-s001.pdf]

Supporting Information

Natural scaffolds with multi-target activity for the potential treatment of Alzheimer´s Disease

Luca Piemontese[1,2,3,*], Gabriele Vitucci[1,2], Marco Catto[1], Antonio Laghezza[1], Filippo Maria Perna[1,3], Mariagrazia Rullo[1], Fulvio Loiodice[1], Vito Capriati[1,3], Michele Solfrizzo[2].

The file includes:

- Figure S1
- Figure S2
- Figure S3
- Figure S4
- Figure S5

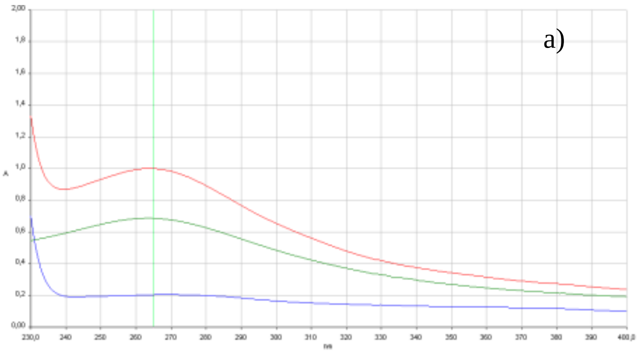

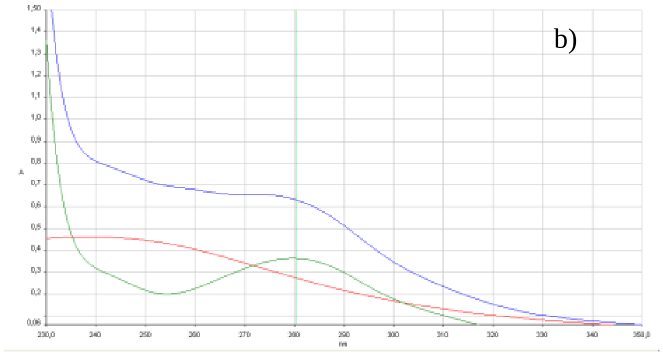

Figure S1a. UV spectra of copper (II) solution (green track), ligand solution (blue track) and copper(II)/ligand 4:1 solution (red track). **Figure S1b.** UV spectra of copper (II) solution (red track), ligand solution (green track) and copper(II)/ligand 4:1 solution (blue track). The experimental conditions are reported in Section 4.5. a) Clioquinol; b) compound **1**.

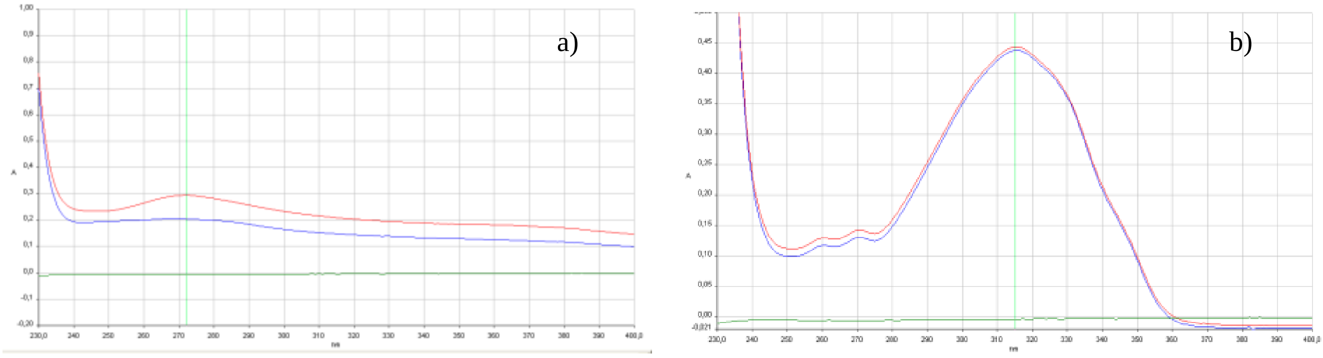

Figure S2. UV spectra of zinc (II) solution (green track), ligand solution (blue track) and copper(II)/ligand 4:1 solution (red track). The experimental conditions are reported in Section 4.5. a) Clioquinol; b) compound **2**.

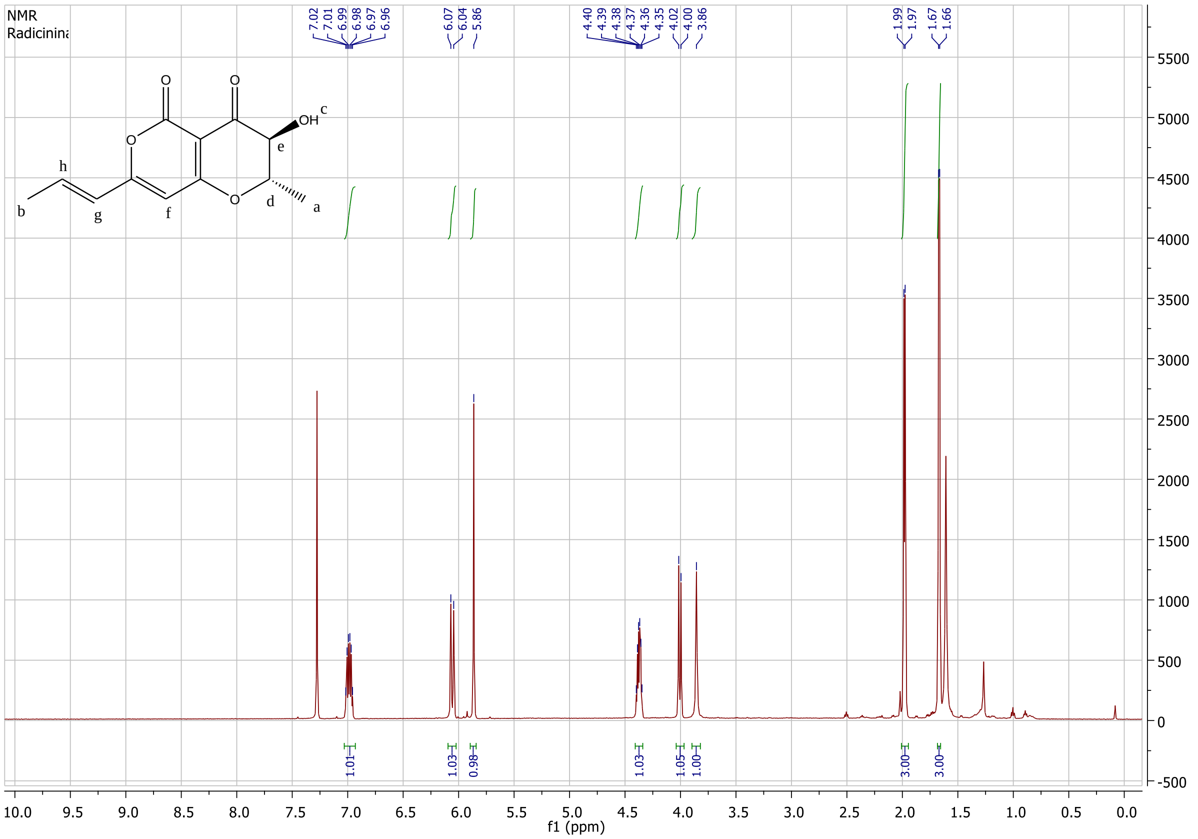

Figure S3. ^{1}H NMR spectrum ($CDCl_3$; 600 MHz) of Radicinin (purity > 97%).

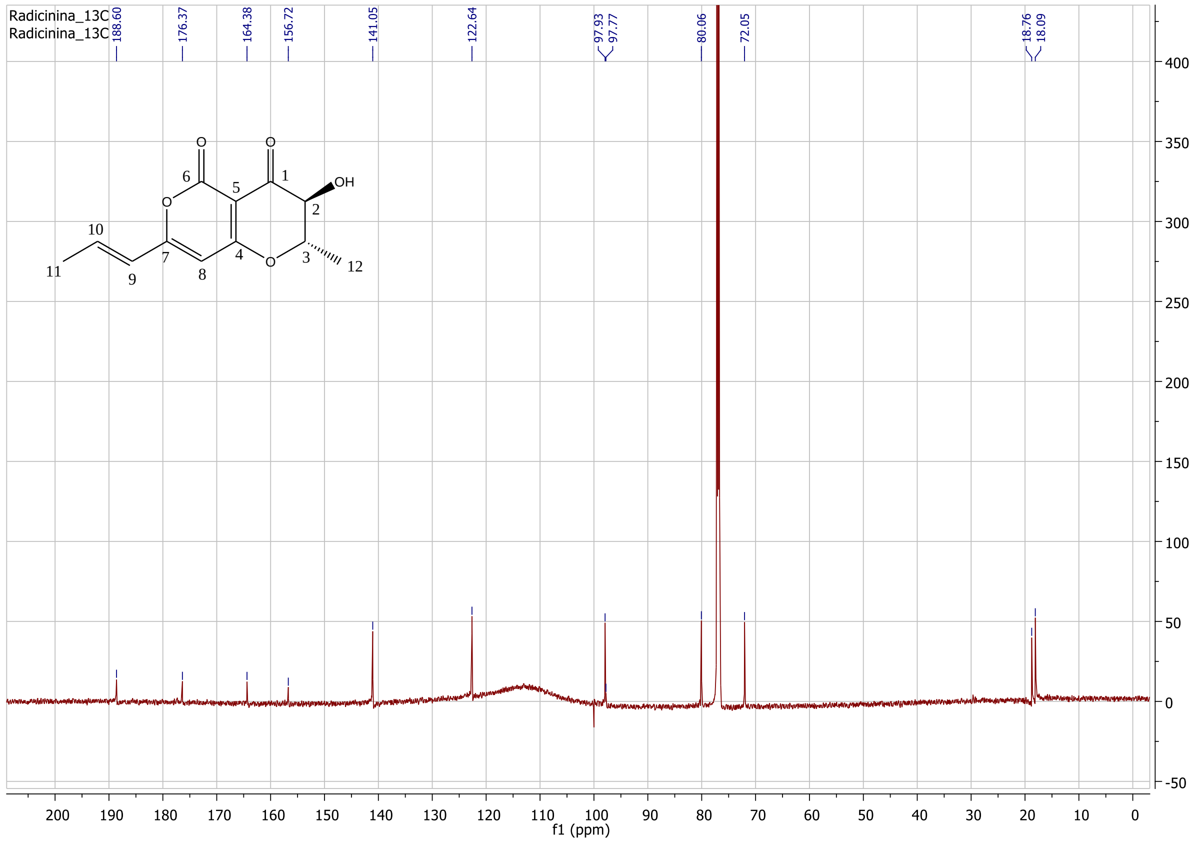

Figure S4. ^{13}C NMR spectrum ($CDCl_3$; 125 MHz) of Radicinin (purity > 97%).

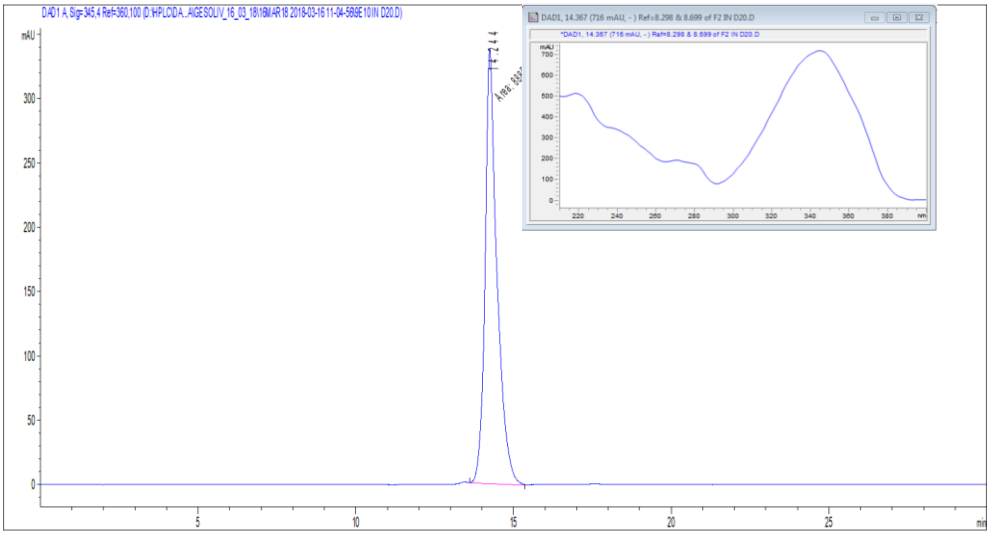

Figure S5. Chromatogram and UV spectrum of Radicinin (purity > 97%). The chromatographic conditions are reported in the text.
